# Playing the complex game of social status in school – a qualitative study

**DOI:** 10.1080/16549716.2020.1819689

**Published:** 2020-10-05

**Authors:** Junia Joffer, Eva Randell, Ann Öhman, Renée Flacking, Lars Jerdén

**Affiliations:** a Department of Epidemiology and Global Health, Umeå University, Umeå, Sweden; b Center for Clinical Research Dalarna-Uppsala University, Falun, Sweden; c School of Education, Health and Social Studies, Dalarna University, Falun, Sweden; d Umeå Centre for Gender Studies, Umeå University, Umeå, Sweden

**Keywords:** Subjective social status, popularity, gendered norms, health, adolescent, intersectionality

## Abstract

**Background:**

Research suggests that social status in school plays an important role in the social lives of adolescents and that their social status is associated with their health. Additional knowledge about adolescents’ understanding of social hierarchies could help to explain inequalities in adolescents’ health and guide public health interventions.

**Objective:**

The study aimed to explore what contributes to subjective social status in school and the strategies used for social positioning.

**Methods:**

A qualitative research design with think-aloud interviews was used. The study included 57 adolescents in lower (7^th^ grade) and upper secondary school (12^th^ grade) in Sweden. Subjective social status was explored using a slightly modified version of the MacArthur Scale of Subjective Social Status in school. Data were analyzed using thematic network analysis.

**Results:**

The participants were highly aware of their social status in school. Elements tied to gender, age, ethnicity and parental economy influenced their preconditions in the positioning. In addition, expectations on how to look, act and interact, influenced the pursue for social desirability. The way these different factors intersected and had to be balanced suggests that social positioning in school is complex and multifaceted.

**Conclusions:**

Because the norms that guided social positioning left little room for diversity, the possible negative impact of status hierarchies on adolescents’ health needs to be considered. In school interventions, we suggest that norms on e.g. gender and ethnicity need to be addressed and problematized from an intersectional approach.

## Background

Social status is closely associated with various health outcomes [1]. This association follows a gradient, i.e. higher status implies better health [2]. A clear association has been demonstrated in different countries, in men and women, in adults and children [2]. For adolescents, however, the association seems to be less consistent; some studies demonstrate an association [3,4], while others show a weak or no association [5,6]. Social status is often studied using objective socioeconomic measures. However, subjective measures that capture people’s perceived standing within a status hierarchy are increasingly used and are regarded as comprehensive measures of social position [7,8]. Singh-Manoux, Marmot and Adler [8] found correlations between subjective and objective measures. Subjective social status was a better predictor of changes in health over time. In adolescents, subjective measures enable the study of adolescents’ social positioning (in contrast to socioeconomic measures, which represent parental social status).

In 2001, Goodman and colleagues [9] developed two youth-specific subjective social status measures in the form of ladders. In one ladder adolescents were asked to assess their position within their school; in the other they were asked to evaluate their families’ position in the society. Subjective social status in school has been associated with self-rated health, physical symptoms, anger, psychological distress, smoking, drinking and body mass index [9,10,11,12,13,14,15,16]. Joffer and colleagues [10] found that the proportion of adolescents with high self-rated health increased with higher rungs on a ladder measuring social status in school, and that boys perceived their own social status as higher than girls did. However, among adolescents in the U.S., girls rated their own status higher than boys did [9]. Status hierarchies in schools could be considered a natural phenomenon [17], but Hiltunen [18] noted that the constant comparison with others, particularly in school, is an important contributing factor to ill health among adolescents.

Popularity is a central aspect of social status in school [19]. In quantitative studies, popularity is often assessed through ‘peer nominations’ in which respondents are asked to nominate peers whom they like/dislike or regard as popular/unpopular [20]. Plenty and Mood [4] found an association between high popularity and better self-rated health. There are several studies on popularity and aggression [21,22,23], showing for example that popularity is positively correlated with proactive aggression both concurrently and over time [23]. Cillessen [24] noted that popularity seems to be a mixture of prosocial and antisocial traits and behaviors. Research on bullying shows that bullies enjoy a high social standing among their classmates [25]. Interviews with elementary schoolchildren in Sweden showed that bullying is partly explained as a struggle for status, power, popularity or friends [26]. Merten [27] found that popular girls in junior high school exerted dominant and mean behaviors. Research suggests an association between popularity and academic difficulties for adolescents who also display aggressive behavior [28].

Hiltunen [18] found that adolescents in Sweden are highly aware of their social status (relative to others) and that of their peers. Qualitative findings from adolescents in the U.S. showed that social status was depicted through physical appearance and brand name clothing, as well as through behavioral aspects, such as hanging out with a popular crowd, playing football or being a cheerleader [29]. Qualitative research on popularity suggests somewhat different determinants for girls and boys: while attractiveness, intelligence and economic resources were central to girls’ popularity, being athletic, tough and disengaged from school were central for boys [30]. Children and adolescents in the U.S. described popular peers as attractive with frequent peer interactions, whilst unpopular peers were described as unattractive, deviant, incompetent and socially isolated [31]. Overall there seems to be a complexity linked to the social construction of popularity [32]. Eder [33] concluded that popular students are not always well liked. Cillessen and Marks [20] denoted an age-difference in the understanding of popularity; while smaller children conceptualized popularity as being well liked, adolescents referred to it as being visible and prestigious. Lundberg [34] emphasizes the need for qualitative studies to gain a better understanding of what subjective social status is, and how to measure it.

As research shows that adolescents’ sense of position in the social hierarchy plays an important role in their lives and influences their health, it is essential to learn more about social status in school. Quantitative research provides an understanding of the importance of age and gender for adolescents’ constructions of status and popularity. There is, however, a need for additional qualitative studies, addressing the complex intersection of various contributing factors. By means of a qualitative approach, adolescents are enabled to express their views on factors that contribute to social status and the various strategies they use when positioning themselves. Such knowledge, developed through the voices of adolescents, is likely to provide important insights when addressing health inequalities and working with health promotion in schools. This study aimed to explore what contributes to subjective social status in school and the strategies used for social positioning.

## Methods

### Study design

A qualitative inductive (emergent) research design with concurrent and retrospective think-aloud interviews with probes and semi-structured interview questions was employed. Think-aloud interviewing is a cognitive interviewing technique used to capture participants’ thoughts as they answer a question (concurrent) or immediately after answering (retrospective). The method seeks to reveal the process of thinking when interpreting and answering a question [35]. A combination of concurrent and retrospective methods has been suggested for producing optimal data quality in think-aloud interviews [36]. Although the primary use of think-aloud interviews is to study the process of thinking when answering survey questions, the richness of the generated data may enable additional use. Thus, in the present study, the ‘think-aloud’ methodology was also a way to facilitate participants to reflect upon the issue of ‘social status in school’.

#### Subjective social status in school

**Figure 1.**
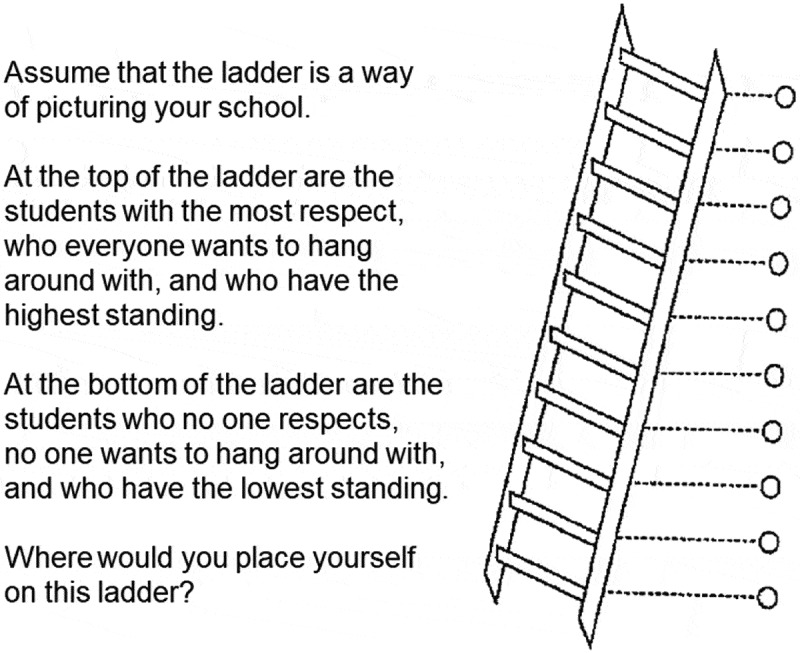
The survey question ‘subjective social status in school’ used in the study, based on Goodman and colleagues [9].

In this study, subjective social status in school was explored through a youth version of the MacArthur Scale of Subjective Social Status, developed by Goodman and colleagues [9]. The question was illustrated by a ladder with ten steps accompanied by an explanatory text which ended with a question (Figure 1). The wording was slightly modified in the present study compared with the original wording [9]. While Goodman and colleagues embedded the term ‘grades’ in their text as a way of describing students with a high position, this term was not included in the Swedish version [10].

### Setting and sample

The study was conducted in a town in the middle of Sweden, which during the years of data collection (2011–2012), had a population of approximately 56 000 inhabitants. The town is representative of the country concerning educational level, income, employment and academic school grades [37]. The public sector covers the main employment in the town, and higher education is available through Dalarna University.

In Sweden, elementary school encompasses 10 years of compulsory schooling and usually starts from the age of six. After compulsory school, most adolescents attend upper secondary school for three additional years. In upper secondary schools, the education is divided into different types of school programs: ‘academic’ programs (preparatory for higher education), ‘vocational’ programs and ‘introductory’ programs (for adolescents who do not have approved grades in Swedish, English and math in compulsory school). In both compulsory school and upper secondary school students are assigned a specific school class with whom they mostly attend the same courses. Both compulsory school and upper secondary schools are free of charge.

In lower secondary school (7^th^ grade, 12–13-year-olds) participants were recruited from two schools. In upper secondary school (12^th^ grade, 17–18-year-olds) participants were recruited from academic, vocational, and introductory school programs in one school. Participants were selected through purposive sampling aiming for maximum variation, taking gender, age, ethnicity and type of school program into consideration. School nurses and class teachers were informed about the study before recruitment. Eleven school classes involving a total of about 275 pupils were approached during class hours (by the first author). Students were provided with both oral and written information about the study and the interviewer. Those who agreed to participate signed a consent form. Students in the 7^th^ grade were also required to obtain informed consent from their parents. The participants were informed about their right to end the interview at any time. After each interview, participants were asked how they felt about being interviewed and information was provided on the possibility of talking to the school nurse/counselor if needed. The study was approved by the Regional Research Ethics Committee at Uppsala University (Dnr 2011–110).

The study included 58 participants, 23 in the 7^th^ grade and 35 in the 12^th^ grade. Because of language barriers and difficulties in understanding the concept of ‘social status’, a boy in 12^th^ grade was excluded from the analysis. Hence the analysis included 57 participants. A few older participants who initially consented to participation changed their minds. They were not asked to explain, but one girl said that she felt stressed about homework. The characteristics of the participants are described in Table 1.

**Table 1.** Characteristics of the study participants.

	Boys n = 28	Girls n = 29
School age		
Lower secondary	10	13
Upper secondary	18	16
Country of birth		
Born in Sweden	24	28
Born outside of Europe	4	1
Subjective social status in school		
1–3	0	0
4	1	4
5	2	5
6	6	6
7	13	5
8	4	5
9	1	3
10	1	1

### Data collection

The overall interview study covered two topics: ‘self-rated health’ (previously reported [38]) and ‘subjective social status’. Two pilot interviews were conducted to test the think-aloud technique and the research protocol. These interviews were not included in the analysis. Before the interviews, the participants completed surveys to provide demographic information. All of the interviews took place in private rooms during school hours. They lasted between 20 and 90 minutes, with a total of 31.5 hours, generating approximately 700 transcribed pages, of which about 350 referred to subjective social status. Swedish was the main language of the interviews, but English was sometimes used for clarification with participants who did not have Swedish as their native language. The interviews were audio-recorded and transcribed verbatim. Transcripts were not returned to participants. The participants were interviewed only once, and the first author performed all interviews with no one else present. By the last few interviews, the researchers were experiencing data saturation in terms of informational redundancy, as similar comments to those in previous interviews were being made by the participants [39]. All participants received a movie ticket for their participation.

The participants were asked neutral questions, such as ‘How do you consider the weather today?’ and ‘What do you think about dogs?’ to practice the think-aloud technique before being asked the research questions (first about ‘self-rated health’, then about ‘subjective social status in school’). When the participants understood how the interview would be conducted, the research questions were presented on a sheet of paper (one paper per question, presented one at a time).

In the concurrent phase of the interview, the participants were instructed to say aloud everything they thought about when answering the question. In the case of silently answering the question a subsequent probe was used: ‘What were you thinking about when answering the question?’ This probe initiated the retrospective phase of the interview. Thereafter, the following probes were used: ‘Did you say everything that you thought about? Did you get a direct feeling of which step [on the ladder] you would choose? Were you sure about your answer?’ Finally, questions were posed to explore further what subjective social status in school comprises and what contributes to different social positions. After performing approximately half of the interviews, one topic emerged more frequently, namely gender differences. Thus, a question on gender was added to the remaining interviews to explore this topic further.

### Data analysis

The interviews were analyzed using thematic network analysis, a method for conducting thematic analysis by creating themes from the qualitative material [40]. The method derives from a hermeneutic tradition and themes are extracted at three different levels: ‘basic themes’, which represent the lowest-order themes derived from the coded data, ‘organizing themes’, which represent categories of basic themes, and ‘global theme/s’ representing the key point/s of the text. In the first stage of the analysis all transcripts were read to form an overall understanding of the content. In the next stage interviews were divided into four groups, and color-coded: younger girls, younger boys, older girls, and older boys. This division made it possible to explore potential differences and similarities between younger and older participants’ ways of describing subjective social status, as well as differences and similarities between boys’ and girls’ descriptions.

After re-reading and coding all the interviews, separate codes were clustered into 10 basic themes. From these basic themes, three organizing themes emerged. The organizing themes were then clustered into one global theme, representing the key point of the text. No software was used for data management. Although some differences between younger and older participants and boys and girls occurred, it was still feasible to summarize their views within one common network. The identified differences between the groups (boys, girls, younger, older) are described within each separate theme in the results.

In the next stage of the analysis the original text was re-read with the aid of the common network. A summary of the themes and patterns characterizing the themes, was described. Finally, the themes were related to the original research question. The first and second authors performed separate coding of all the data, discussed the findings together and created the network. While the first and second authors conducted most of the thematic network analysis, all authors discussed the content of the interviews, took part in the analytical phase, contributed to the interpretation of the text and the creation of the network. Participants were not asked to provide feedback on the findings.

#### Integrating a gender perspective

Connell [41] stresses the importance of addressing gender issues in health research from a ‘relational theory of gender’. This theory implies looking beyond a categorical way of thinking in which masculinity and femininity are seen as opposites. A relational theory acknowledges gender as an active social process that generates health consequences. From this perspective, gender is regarded as multidimensional and differences within gender categories are acknowledged. In the interviews, participants defined themselves as either a boy or a girl. In the analysis, however, we sought to acknowledge active social processes and to explore how social norms reflected the participants’ experiences. The ways in which masculinities and femininities were constructed within groups of boys and girls were also explored.

## Results

### Playing the complex game of social status

**Figure 2.**
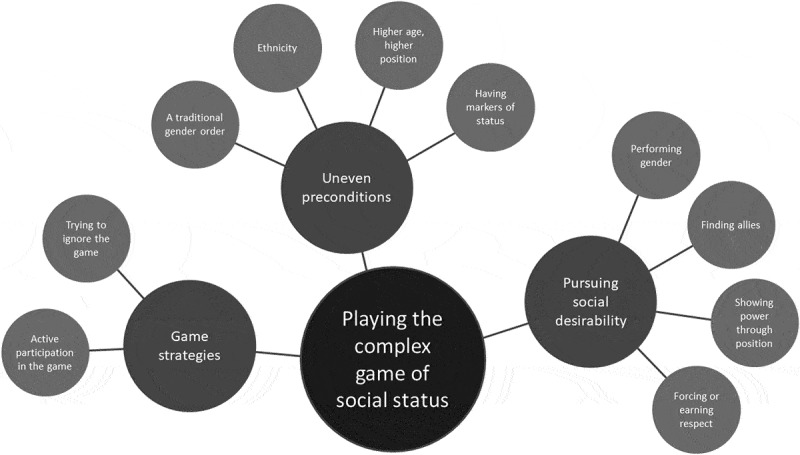
Network of themes describing factors and strategies that influence adolescents while playing the complex game of social status in school.

The social positioning in school has similarities to a complex game. While most participants presented an active participation, some were trying to ignore the game. Participants acknowledged various factors that interacted and contributed to the positioning. Some were, by character, largely fixed as they derived from gender, ethnicity, age and parents’ economy, whereas others related to more active choices of how to look, act and interact. Figure 2 depicts characteristics and game strategies that influenced them while *playing the complex game of social status*.

#### Game strategies

Social comparisons were mostly regarded as a natural phenomenon and participants expressed *active participation in the game*. Participants were highly aware of the game of social status and could rather easily position themselves and other students on a ladder of social status. Girls and younger participants expressed a more obvious need to follow prevailing norms and attempted to play the game by the rules. In the social hierarchy those at the top set the agenda and their opinions influenced others. A higher position seemed to be the result of conscious and active engagement. As one 12^th^ grader said, ‘I’m sure those on the top are really smart, really good looking and very cool, but, I also think it’s a conscious choice, they strive to become as popular as possible’ (Boy, 12^th^ grade).

Some participants were active players, yet conveyed a less pronounced engagement. They were aware of the game and the rules but expressed satisfaction over a less demanding position to play it safe. One girl explained, ‘You don’t want to be at the bottom, but you don’t want to be on the top either because it may be demanding’ (Girl, 12^th^ grade). Statements such as ‘I try to be humble’ or ‘I regard everyone as equal’ were made to justify such a strategy. Safe play was often equated with having a position in the middle of the ladder, which enabled socialization with peers on both ends of the ladder. A few participants had a hard time accepting the social status ladder, but acknowledged its presence. One boy in the 12^th^ grade who chose the fifth step on the ladder exemplified this feeling.

Of course, you can see this ladder everywhere, really. You want to see yourself as a bit higher than you really are. I have a hard time accepting the ladder. But as I said, you see it all the time. (Boy 12^th^ grade)

Although most participants recognized the social ladder in school, a few of the older participants actively distanced themselves from the social game, *trying to ignore the game*. In this respect, ignoring the social status ladder seemed to represent a protest against social hierarchies in school, ‘Now I don’t know what to answer, because I don’t think there is such a thing here. There are those who are seen and heard more than others, but I don’t respect them more’ (Girl, 12^th^ grade).

#### Uneven preconditions

Gender, ethnicity, age and parents’ economy affected the participants’ prerequisites in the game. These factors intersected and contributed to the positioning. *A traditional gender order* related to the power dynamics between boys and girls, and within groups of boys and girls, and resulted in different positions of opportunity. The boys’ advantage was discussed through reflections on patriarchal gender norms. One young girl recognized boys’ higher position on the ladder, ‘The most popular boys [points at the top of the ladder] and the less popular boys and the most popular girls [points a little lower on the ladder]’ (Girl, 7^th^ grade). For girls, having a popular boyfriend facilitated a higher position, ‘Maybe you are dating a popular boy; then you also become popular’ (Girl 7^th^ grade). In comparison with boys, girls were more prone to compare themselves with other girls.

The notion of different *ethnicities* created gaps between diverse cultural groups. For instance, some of the older participants recognized being white-skinned and mastering the Swedish language as more highly valued. The problem of social distance of peers from cultural minorities was highlighted, as one boy remarked, ‘I am not with Swedes, we do not speak with each other and I have no Swedish friends’ (Boy, 12^th^ grade). Only looking in a way that differed from the ethnic norm implied a higher risk for exclusion and being bullied: ‘So, I’ve always been bullied, since I was three years old, because of the color of my skin and everything’ (Girl, 12^th^ grade).

When different age groups were compared, *higher age* typically implied a *higher position* on the social ladder. Younger participants mostly reflected the superiority of older peers: ‘If you position yourself in relation to the whole school, then 9^th^ graders see themselves as the best’ (Boy, 7^th^ grade). Older participants considered status markers (e.g. a driver’s license) that came with being older as they represent power and evoke admiration.

Participants’ thoughts and reflections about their parents’ economy were made in terms of *having markers of status*. Having money enabled buying status markers such as designer clothes. Thus, status markers facilitated a higher position in the social hierarchy. Not being able to buy the most recent marker could become a problem.

A while back, everyone was supposed to have an Adidas bag, one of those squared bags. I really wanted one because the really cool ones had one. So then, I got it for Christmas. When I came to school I became so proud, because I had one. Everyone had one. But there was a girl who didn’t and that became difficult for her. Almost no one wanted to talk to her. Imagine how hard it must have been for her, not to get such a bag. (Girl, 7th grade)

#### Pursuing social desirability

The way a person looked, acted and interacted with others in school revealed that person’s position on the ladder. One girl exemplified some of the elements that contributed to social desirability. She said, ‘There are a lot of aspects to consider. It depends on how you dress, what school program you go to, whether you are a boy or a girl. I would place myself rather high’ (Girl, 12^th^ grade).

Both younger and older participants reflected on *performing gender*. Participants reported that they were expected to look and act in a certain way depending on their gendered belonging. One girl in the 7^th^ grade described socially desirable appearances and the higher demands that came from being a girl.

Boys should have a brand sweatshirt, nice jeans or chinos. Not everyone can afford it, so those who can become higher ranked. With the girls, it’s so much. You can’t have sweatpants, that’s just wrong. You should have leggings or jeans. It’s so much. I mean the hair and everything. Shoes – it’s so very much. (Girl, 7^th^ grade)

Prevailing gender norms guided the valuing of different ideal characteristics. Popular boys were often described as ‘being strong’, ‘athletic’ or ‘having high self-esteem’, whereas girls often were valued by factors such as ‘nice clothes’, ‘nice hair’ or ‘being cute’. One boy explained, ‘You’re well respected, and kind of big and strong […] for girls it is maybe, I don’t know, you shouldn’t have any prejudices, but maybe the nicest clothes, the nicest makeup, the one who looks the best’ (Boy, 7^th^ grade). It was more socially acceptable for boys to be confident and show a tough attitude; girls on the other hand were expected to do things with moderation. Girls sometimes underlined that ‘mature’ boys were the popular ones. Good grades and achievements in school were used to describe high-positioned girls. Norm-compliance was important for a higher positioning, which was most prominent in girls and younger participants. One participant described different looks for boys and girls and how they would reveal their social status position.

Popular students are those wearing a cap, having rather short hair and some kind of hoodie. Not black – black hoodies are for nerds. Girls with far too little clothes, who walk around freezing are popular. Let’s see – if you look like me – torn jeans and a poorly fitting t-shirt, then you’re rather low [on the social status ladder]. (Boy, 12^th^ grade)

The process of positioning oneself on the social status ladder sometimes included the act of striving for a higher position while not losing one’s current position. Highly ranked students could influence norms, but there was a fine line between affecting norms and the chance of risking their reputation. Girls appeared to be particularly targeted in this respect. As one boy stated, ‘Girls are at risk of losing their reputation very, very quickly. It might be because she’s trash-talking other people or maybe because she’s sleeping with guys’ (Boy, 12^th^ grade). When reflecting on sexuality, older boys and girls expressed similar views on how they were expected to perform and behave.

If a boy has sex with several girls, then he’s very manly, he’s popular, whereas if a girl has sex with several boys, then she becomes known as a whore … then you’re promiscuous, while if a boy does it, he becomes instantly highly ranked, a real stud. (Girl, 12^th^ grade)

In the social game participants were conscious of whom they ‘hung out’ with and strived to *find allies*. They expressed making conscious choices. As one girl said, ‘If I were to become friends with those in my class who have it [lower status], I would also get it’ (Girl, 7^th^ grade). Their group belonging was highly valued and the importance of having friends was emphasized, particularly by the younger participants. The term ‘popularity’ was used to describe top positioned peers and the number of friends was a way of assessing popularity. One girl remarked, ‘I’m somewhere below the middle because I’m a geek. Still I have friends. So I’m not at the bottom and really not at the top’ (Girl, 7^th^ grade). Status position was linked to both the individual and the group. An individual could have a high position and have many friends within the closest social grouping, but a lower position if the group was ranked low in the school. One boy noted, ‘In my school class I’m one of the highest-ranking students. However, since my class is rather lowly placed in school, I’d place myself low: the fourth step from the bottom’ (Boy, 12^th^ grade). Among older participants, the type of school program influenced the social position. Academic school programs were higher valued compared to vocational or introductory programs. Participants who belonged to groups that were perceived as having lower social status, e.g. a less valued school program or a cultural minority, often expressed a strong sense of unity in the group, which served as a protective shield. Many participants seemed to regard their closest group belonging as more important than their overall position in the school.

The participants’ way of acting in the school space revealed their positions, which implies that they *showed power through position*. One 7^th^ grader explained, ‘Someone standing in the middle talking a lot, they may be at the top. Then those who stand around and listen, they’re in the middle. And then those who sit by themselves at the table are down here’ (Boy, 7^th^ grade).

The duality of having a top position was reflected through *forcing or earning respect*. Respect was sometimes described as something that high-ranked students had forced others to give them, and had not truly earned, i.e. ‘fake-respect’. One participant made the following comment related to bullying, ‘Those who bully get one kind of respect, you don’t argue with them. But I don’t respect them for what they do; they don’t get my respect’ (Boy, 12^th^ grade). Attractive students were assigned the top positions, but this did not automatically imply that they were the most respected. Respect was also considered a positive attribute, e.g. when describing oneself as a good person and a good friend, or when showing respect for others: ‘Behaves in a good way, also in class, and doesn’t disturb others’ (Boy, 7^th^ grade). Participants used both the terms ‘respect’ and ‘popularity’ when describing top-positioned peers. One girl in the 12^th^ grade explained the complex meaning of being on the top of the ladder.

Some become popular because they have money; some because you’re afraid of them or because you don’t want to become their enemy. Or it’s because they’re very nice people who you look up to. (Girl, 12^th^ grade)

## Discussion

The participants’ narratives revealed that subjective social status in school is complex and multifaceted. Three main findings emerged from the voices of the adolescents: most participants were active in the social game and well aware of their position; participants’ gender, age, ethnicity and parents’ socioeconomic status set their preconditions in the social game; and expectations about how to look, act and interact influenced the participants’ pursuit of social desirability. These main findings are discussed in detail below.

### Confirming social hierarchies in school

In this study, participants employed different strategies in the process of social positioning. Most participants confirmed the existence of a social ladder in school and described their active participation. Hiltunen [18] posits the existence of a social game and that adolescents constantly compare themselves with others. Although Fournier [17] advocates that status hierarchies in schools could be considered a natural phenomenon, Hiltunen argues that the constant need to uphold and defend one’s position and the exposure to social-evaluative threats suggest that social hierarchies can be a source of ill health [18]. Garandeau, Lee and Salmivalli [42] identify additional negative factors based on the association between higher levels of classroom status hierarchy (popularity and likeability) and bullying, highlighting the importance of a shared balance of power in the classroom.

### Uneven preconditions in the game

Because boys were described to hold the top positions, a traditional gender order was apparent. The possession of top positions, however, did not apply to all boys. As previously described by Connell [43], hegemonic masculinity (i.e. men’s dominance in society) is not uniform, but rather facilitated by subordinated and marginalized masculinities. Descriptions of boys as tall, muscular and confident indicated that top positioned boys were those who fitted the hegemonic boy norm. Courtenay [44] describes negative impacts on health when performing hegemonic constructions of masculinity. In the present study, possible health risks of the hegemonic boy norms need to be considered for all boys, both norm compliant and subordinated boys. Norm-compliant boys may achieve a top position in the social game; however, because vulnerability is not socially desirable, such norms also imply a health risk because they may hinder these boys from seeking help when experiencing poor health. However, previous quantitative findings show that higher status is associated with better self-rated health [10]. Goodman and colleagues [9] found associations between social status in school and global self-esteem. The combined findings point towards both risks and benefits associated with possessing a high position in the social hierarchy.

The traditional gender order relates to the subordination of femininities in relation to dominant masculinities, referred to as ‘emphasized femininities’ [45]. With Sweden scoring among the highest on the Gender Development Index [46] it is troublesome that this traditional view of gender existed to the extent it did among the participants in the current study. Because adolescents are the future parents and labor force, the norms and values expressed by this group matter greatly to society. Negative health consequences for girls due to subordination need to be considered, in terms of exposure to gendered violence and sexual harassment [47]. Although a hegemonic gender order was acknowledged, participants’ reflections about the unfairness of masculine dominance indicated a degree of resistance towards these structures. This attitude was presented by expressing disdain towards the traditional gender order.

The participants’ reflections about status markers and parental economy indicate that traditional socioeconomic markers influenced their social position. Wealthy parents facilitated the purchasing of status markers. Younger, in comparison with older participants, discussed status markers more often as a way of following prevailing norms on how to look. Previous research has shown that social status among peers can be acquired by the possession of material resources observable to others [18,29]. It has previously been concluded that affluence may be especially central for girls’ social status and popularity as they seek to stay in touch with the latest fashions [19,30,33]. Rysst [48] concluded that popularity among girls is connected to consumption, which may have negative consequences for immigrant girls because they often live in low-income families.

### Pursuing social desirability

The participants’ pursuit of social desirability related to their strategies for social positioning. Participants described narrow norms and expectations on how to look, act and interact. Norms, which represent what is socially desirable, enable social interaction and offer a guide on how to act in a particular setting. However, norms are also limiting for those that do not fit the description of what is socially desirable and whose identity is far from the norm [49]. Because norms are not separated from each other, an intersectional perspective provides an understanding of how norms reinforce one another [50,51,52]. In the present study, participants characterized norm compliance as being perceived as male, heterosexual, tall, muscular, white-skinned and having parents who could afford to buy status markers. Those adolescents were positioned on the very top of the social ladder. If one of these factors were to change, so would the individual’s position on the ladder, indicating that norms interact and reinforce one another. Read, Francis and Skelton [53] have previously reported that adolescents recognize the need to ‘act like everyone else’ and ‘play the game’ in order to ‘fit in’ and become popular. Narrow and excluding norms are worrisome as they are an obstacle to diversity. Yet, while norms are bound to time, context and situation, they can also change. School is a context in which norms are both reinforced and challenged [49]. Because school prepares children and adolescents for adult life, it is an important arena for the facilitation of change.

Gender differences in the performance of social status were found in the valuing of the body and expectations on how to look and act. Girls had to follow more rules in this regard and appeared to be more targeted and vulnerable to the judgment of others. Older participants discussed the reproduction of traditional gender norms in which girls’ sexuality was restricted and put under moral judgement. While boys gained status from having many sexual partners, such behavior lowered the status of girls. Sexualization in school and the reproduction of a gender order have been described elsewhere [49,54]. Gillander Gådin and Hammarström [54], for instance, see the school environment as an important context for sexualized behaviors, as boys sought power and dominance over girls. Wiklund [55] recognizes the presence of social rankings in parallel arenas in the lives of young women in that the body (both inner experiences and outer explorations) is central in the identity-making process. Wiklund found that young girls are well aware of their bodies as a central feature of their performing of social status [55]. Traditional gendered expectations are enabled through the heterosexual normativity [56], which affects and limits the behaviors of both boys and girls [49], but also non-binary adolescents. Strömbäck and colleagues [57] note that young women face an exhausting and draining self-evaluating circle because they are expected to handle both the historical position of subordination and a discourse of successful femininity. Our study found that girls had to balance multiple factors regarding e.g. sexuality, physical appearances, and achievements in school, to become socially desirable. The possible negative impact on girl’s health when balancing multiple factors (performing successful femininity), needs to be considered.

The school program was a factor influencing the older participants’ positioning. Academic school programs were valued higher than vocational and introductory programs. This finding is consistent with previous research [58,59]. Our findings indicate that the influence of school program should be understood through a system of interactions between sociocultural categorizations, norm-producing discourses and power relations, as suggested in intersectional theory [60,61]. Belonging to an academic school program did not on its own facilitate a high position. The interlinkage with other categorizations such as gender identity, sexuality, ethnicity, age and socioeconomic status, also needed to be considered. In accordance with ‘intersectional gender pedagogy’ the findings of the present study underline the need to reflect on the consequences of differences, power and inequalities in the classroom, as previously proposed by Lykke [61]. By making differences within the classroom visible, norms that create inequality and exclusion are counteracted. Lykke suggests the use of ‘transversal dialogue’ to explore the landscape of power and norms within classrooms; a tool aiming to reflect on one’s own intersectional rooting, and the intersectional rooting of others. As intersections were highly visible in the present study, the use of transversal dialogue may be one way of addressing inequalities in school.

Participants described the importance of friends and the closest group belonging, which served as a protective shield. Research shows that the importance of peer relationships increases through childhood and adolescence in which peer acceptance and rejection are important determinants [62]. Being accepted and having friends is a basis for wellbeing [63,64]. A low position on the ladder seemed to be compensated by the closest group belonging and participants described forming alliances as a way to resist the pressure from others. Younger participants often reflected on the importance of having friends. The relevance of peer relationships in the school class for health development across the life-course has previously been recognized [65]. Accordingly, in the school environment the power of friends and the closest group belonging should be underlined.

When reflecting on the top-positioned students, the terms ‘respect’ (indicated in the question about subjective social status) and ‘popularity’ were used. Participants expressed that top-positioned peers not always were the most respected. Studies on ‘peer status’ and ‘popularity’ [32,33,66] are mindful of the complexity of these terms as the most popular students are not always the most liked. Closson [32] has previously found that adolescents’ perceptions of popularity differ based on their own popularity, with adolescents who were perceived as popular describing popularity in more positive terms than average and unpopular adolescents. Thus, both previous research and our study reveal complexity linked to the terms ‘respect’ and ‘popularity’.

### Methodological considerations

When referring to credibility, the study sample was relatively large and included boys and girls from different socioeconomic backgrounds, schools and educational programs in the 12^th^ grade (academic, vocational and introductory school programs). Despite efforts to recruit from the introductory program, the study failed to include adolescents with the lowest social status positions (steps 1–3). It is possible that other ways of approaching students would be more successful in recruiting participants with the lowest ratings. Still, we believe that participants on the lower half (step 1–5) of the ladder (21% of the participants) were sufficiently represented to gain different perspectives on the subject. Compared with quantitative studies [10,11], our study had a similar or even higher representation of lower-status participants. The study was performed in Sweden, a high-income country ranking high on both the Human Development Index and the Gender Development Index [46], suggesting that the transferability primarily relates to similar cultures.

The concurrent and retrospective think-aloud technique with probes and semi-structured questions allowed us to explore subjective social status in greater depth, and gave participants who were not as verbally skilled the opportunity to provide richer descriptions. The separate coding of all the interview transcripts and the interdisciplinary contributions from the co-authors collectively increase the trustworthiness of the study. The division of participants into different groups in the analysis (girls, boys, younger, older) facilitated capturing the similarities and differences in the participants’ experiences and beliefs.

In qualitative research the researchers’ theoretical foundation and conceptual pre-understandings are used and made transparent through reflexive reasoning. The research group was interdisciplinary (schooled within the areas of public health, social work, gender and feminist studies, pediatrics and family medicine), which enriched the analysis as different perspectives were considered.

## Conclusions

The participants were well aware of the different factors that influenced them in the social game. Because the norms that guided social positioning left little room for diversity, the possible negative impact of status hierarchies on adolescents’ health needs to be considered. In school interventions, when striving for equality, we suggest that factors such as gender, ethnicity, sexuality, expectations about how to look and act etc. need to be addressed and problematized using an intersectional approach. By incorporating an understanding of such factors as coexisting and intertwined and by acknowledging that adolescents are not a homogenous group, the norms that create hierarchies and inequalities may be counteracted.

## Data Availability

Data are stored at the Center for Clinical Research in Dalarna County. Because the data consist of interviews with sensitive personal information, data will not be shared.

## Paper context

The interest in subjective social status has increased, especially as research indicates an association with adolescents’ health. This study explores factors that contribute to social status in school, as well as strategies used for positioning. Our findings revealed that adolescents confirmed a social ladder in school and that traditional norms related to for example gender and ethnicity influenced their positioning. School represents a key setting through which norms can be addressed and change facilitated.

## References

[1] Adler NE, Ostrove JM. Socioeconomic status and health: what we know and what we don’t. Ann N Y Acad Sci. 1999;896:3–12. 10681884 10.1111/j.1749-6632.1999.tb08101.x

[2] Marmot M. Social determinants of health inequalities. Lancet. 2005;365:1099–1104. 15781105 10.1016/S0140-6736(05)71146-6

[3] Boynton-Jarrett R, Ryan LM, Berkman LF, et al. Cumulative violence exposure and self-rated health: longitudinal study of adolescents in the United Status. Pediatrics. 2008;122:961. 18977974 10.1542/peds.2007-3063 PMC8679309

[4] Plenty S, Mood C. Money, peers and parents: social and economic aspects of inequality in youth wellbeing. J Youth Adolescence. 2016;45:1294–1308. 10.1007/s10964-016-0430-5 PMC4901095 26847325

[5] Siahpush M, Singh G. A multivariate analysis of the association between social class of origin and current social class with self‐rated general health and psychological health among 16‐year‐old Australians. Aust N Z J Med. 2000;30:653–659. 11198572 10.1111/j.1445-5994.2000.tb04359.x

[6] Hagquist C. Health inequalities among adolescents - the impact of academic orientation and parents’ education. Eur J Public Health. 2006;17:21–26. 16777839 10.1093/eurpub/ckl087

[7] Euteneuer F. Subjective social status and health. Curr Opin Psychiatry. 2014;27:337–343. 25023883 10.1097/YCO.0000000000000083

[8] Singh-Manoux A, Marmot MG, Adler NE. Does subjective social status predict health and change in health status better than objective status? Psychosom Med. 2005;67:855–861. 16314589 10.1097/01.psy.0000188434.52941.a0

[9] Goodman E, Adler NE, Kawachi I, et al. Adolescents’ perceptions of social status: development and evaluation of a new indicator. Pediatrics. 2001;108:e31. 11483841 10.1542/peds.108.2.e31

[10] Joffer J, Flacking R, Bergström E, et al. Self-rated health, subjective social status in school and socioeconomic status in adolescents: a cross-sectional study. BMC Public Health. 2019;19:785. 31221114 10.1186/s12889-019-7140-3 PMC6587278

[11] Diehl K, Hoebel J, Sonntag D, et al. Subjective social status and its relationship to health and health behavior: comparing two different scales in university students. Int J Adolesc Med Health. 2017;31. DOI:10.1515/ijamh-2017-0079 28841574

[12] Åslund C, Leppert J, Starrin B, et al. Subjective social status and shaming experiences in relation to adolescent depression. Arch Pediatr Adolesc Med. 2009;163:55–60. 19124704 10.1001/archpedi.163.1.55

[13] Sweeting H, Hunt K. Adolescent socio-economic and school-based social status, health and well-being. Soc Sci Med. 2014;121:39–47. 25306408 10.1016/j.socscimed.2014.09.037 PMC4222198

[14] Sweeting H, Hunt K. Adolescent socioeconomic and school-based social status, smoking, and drinking. J Adolesc Health. 2015;57:37–45. 26095407 10.1016/j.jadohealth.2015.03.020 PMC4510202

[15] Finkelstein DM, Kubzansky LD, Goodman E. Social status, stress, and adolescent smoking. J Adolesc Health. 2006;39:678–685. 17046504 10.1016/j.jadohealth.2006.04.011

[16] Lemeshow AR, Fisher L, Goodman E, et al. Subjective social status in the school and change in adiposity in female adolescents: findings from a prospective cohort study. Arch Pediatr Adolesc Med. 2008;162:23–28. 18180408 10.1001/archpediatrics.2007.11

[17] Fournier MA. Adolescent hierarchy formation and the social competition theory of depression. J Soc Clin Psychol. 2009;28:1144–1172.

[18] Hiltunen L. Lagom perfekt: erfarenheter av ohälsa bland unga tjejer och killar [The pursuit of restrained perfection: experiences of ill health among adolescent girls and boys]. Lund: Arkiv förlag; 2017.

[19] Adler PA, Adler P. Peer power: preadolescent culture and identity. New Brunswick, NJ: Rutgers Univ. Press; 1998.

[20] Cillessen AHN, Marks PEL. Conceptualizing and measuring popularity. In: Cillessen AHN, Schwartz D, Mayeux L, editors. Popularity in the peer system. New York: Guilford Press; 2011. p. 25–56.

[21] Cillessen AH, Mayeux L. From censure to reinforcement: developmental changes in the association between aggression and social status. Child Dev. 2004;75:147–163. 15015681 10.1111/j.1467-8624.2004.00660.x

[22] Rose AJ, Swenson LP, Waller EM. Overt and relational aggression and perceived popularity: developmental differences in concurrent and prospective relations. Dev Psychol. 2004;40:378–387. 15122964 10.1037/0012-1649.40.3.378

[23] Stoltz S, Cillessen AH, van den Berg YH, et al. Popularity differentially predicts reactive and proactive aggression in early adolescence. Aggress Behav. 2016;42:29–40. 26299476 10.1002/ab.21603

[24] Cillessen AHN. Toward a theory of popularity. In: Cillessen AHN, Schwartz D, Mayeux L, editors. Popularity in the peer system. New York: Guilford Press; 2011. p. 273–299.

[25] Juvonen J, Graham S, Schuster MA. Bullying among young adolescents: the strong, the weak, and the troubled. Pediatrics. 2003;112:1231–1237. 14654590 10.1542/peds.112.6.1231

[26] Thornberg R. Schoolchildren’s social representations on bullying causes. Psychol Sch. 2010;47:311–327.

[27] Merten DE. The meaning of meanness: popularity, competition, and conflict among junior high school girls. Sociol Educ. 1997;70:175–191.

[28] Schwartz D, Gorman AH. The high price of high status: popularity as a mechanism of risk. In: Cillessen AHN, Schwartz D, Mayeux L, editors. Popularity in the peer system. New York: Guilford Press; 2011. p. 245–270.

[29] Sweet E. “If your shoes are raggedy you get talked about”: symbolic and material dimensions of adolescent social status and health. Soc Sci Med. 2010;70:2029–2035. 20363543 10.1016/j.socscimed.2010.02.032

[30] Rose AJ, Glick GC, Smith RL. Popularity and gender: the two cultures of boys and girls. In: Cillessen AHN, Schwartz D, Mayeux L, editors. Popularity in the peer system. New York: Guilford Press; 2011. p. 103–122.

[31] LaFontana KM, Cillessen AH. Children’s perceptions of popular and unpopular peers: a multimethod assessment. Dev Psychol. 2002;38:635–647. 12220043 10.1037//0012-1649.38.5.635

[32] Closson LM. Status and gender differences in early adolescents’ descriptions of popularity. Soc Dev. 2009;18:412–426.

[33] Eder D. The cycle of popularity: interpersonal relations among female adolescents. Sociol Educ. 1985;58:154–165.

[34] Lundberg J. Social status: a state of mind? [Doctoral thesis]. Linköping: Linköping University; 2008.

[35] Presser S, Couper MP, Lessler JT, et al. Methods for testing and evaluating survey questions. Public Opin Q. 2004;68:109–130.

[36] Sudman S, Bradburn NM, Schwarz N. Thinking about answers: the application of cognitive processes to survey methodology. San Francisco: Jossey-Bass Publishers; 1996.

[37] Public Health Agency of Sweden. Kommunfaktablad [Facts about municipalities] [Internet]. [cited 2019 830]. Available from: https://www.folkhalsomyndigheten.se/kommunfakta/

[38] Joffer J, Jerdén L, Öhman A, et al. Exploring self-rated health among adolescents: a think-aloud study. BMC Public Health. 2016;16:156. 26880571 10.1186/s12889-016-2837-z PMC4754811

[39] Sandelowski M. Theoretical saturation. In: Given LM, editor. The SAGE encyclopedia of qualitative research methods. Vol. 2. Thousand Oaks: Sage; 2008. p. 875–876.

[40] Attride-Stirling J. Thematic networks: an analytic tool for qualitative research. Qual Res. 2001;1:385–405.

[41] Connell R. Gender, health and theory: conceptualizing the issue, in local and world perspective. Soc Sci Med. 2012;74:1675–1683. 21764489 10.1016/j.socscimed.2011.06.006

[42] Garandeau CF, Lee IA, Salmivalli C. Inequality matters: classroom status hierarchy and adolescents’ bullying. J Youth Adolesc. 2014;43:1123–1133. 24129884 10.1007/s10964-013-0040-4

[43] Connell R. Gender in world perspective. 2nd ed. Cambridge: Polity; 2009.

[44] Courtenay WH. Constructions of masculinity and their influence on men’s well-being: a theory of gender and health. Soc Sci Med. 2000;50:1385–1401. 10741575 10.1016/s0277-9536(99)00390-1

[45] Connell RW. Masculinities. Berkeley, CA: University of Califonia Press; 1995.

[46] Human Development Report Office Team. Human development data (1990–2018) [Internet]. 2017 [cited 2019 923]. Available from: http://www.hdr.undp.org/en/data

[47] Ellsberg M, Arango DJ, Morton M, et al. Prevention of violence against women and girls: what does the evidence say? Lancet. 2015;385:1555–1566. 25467575 10.1016/S0140-6736(14)61703-7

[48] Rysst M. Popularity, gender, and social inclusion among girls in ethnically diverse contexts in Norway. Int J Child Fam Stud. 2020;11:45–69.

[49] Martinsson L, Reimers E. Skola i normer [School in norms]. 2nd ed. Malmö: Gleerup; 2014.

[50] Glenn EN. Racial ethnic women’s labor: the intersection of race, gender and class oppression. Rev Radic Political Econ. 1985;17:86–108.

[51] Hankivsky O. Intersectionality 101. The Institute for Intersectionality Research Policy, SFU; 2014:1–24.Available from: https://www.researchgate.net/publication/279293665_Intersectionality_101

[52] Hankivsky O. Intersectionality 101. The Institute for Intersectionality Research & Policy, SFU; 2014:1–34. Available from: https://www.researchgate.net/publication/279293665_Intersectionality_101

[53] Read B, Francis B, Skelton C. Gender, popularity and notions of in/authenticity amongst 12‐year‐old to 13‐year‐old school girls. Br J Sociol Educ. 2011;32:169–183.

[54] Gådin KG, Hammarström A. “We won’t let them keep us quiet … gendered strategies in the negotiation of power-implications for pupils’ health and school health promotion. Health Promot Int. 2000;15:303–311.

[55] Wiklund M. Close to the edge. Discursive, embodied and gendered stress in modern youth [Doctoral thesis]. Umeå: Umeå University; 2010.

[56] Tolman DL, Davis BR, Bowman CP. “That’s just how it is”: a gendered analysis of masculinity and femininity ideologies in adolescent girls’ and boys’ heterosexual relationships. J Adolesc Res. 2015;31:3–31.

[57] Strömbäck M, Formark B, Wiklund M, et al. The corporeality of living stressful femininity: a gender-theoretical analysis of young Swedish women’s stress experiences. Young. 2014;22:271–289.

[58] Sandell A. Utbildningssegregation och självsortering - Om gymnasieval, genus och lokala praktiker [Educational segregation and self-sorting - Choices of upper-seconday school education, gender and local practices] [Doctoral thesis]. Malmö: Malmö University; 2007.

[59] Panican A. Väljer unga fel? – grundskoleelevers attityder till gymnasievalet [Does adolescents choose wrong? - elementary school childrens’ attitudes about upper secondary school programs]. Lund: Lund University and Ratio; 2015. (Report No.: 19).

[60] Glenn EN. Racial ethnic women’s labor: the intersection of race, gender, and class oppression. In: Blumberg RL, editor. Gender, family, and economy: the triple overlap. Sage focus editions. Vol. 125. Thousand Oaks, CA: Sage Publications, Inc; 1991. p. 173–201.

[61] Lykke N. Intersectional gender pedagogy. In: Lundberg A, Werner A, editors. Gender studies education and pedagogy. A series on gender studies. Gothenburg: Swedish Secretariat for Gender Research; 2014. p. 14–17.

[62] Sentse M, Kretschmer T, Salmivalli C. The longitudinal interplay between bullying, victimization, and social status: age‐related and gender differences. Soc Dev. 2015;24:659–677.

[63] Flacking R, Jerdén L, Bergström E, et al. ‘In or out’—on the dynamic between acceptance and rejection and its influence on health in adolescent girls. Young. 2014;22:291–303.

[64] Randell E, Jerdén L, Öhman A, et al. Tough, sensitive and sincere: how adolescent boys manage masculinities and emotions. Int J Adolesc Youth. 2016;21:486–498.

[65] Almquist Y. A class of origin: the school class as a social context and health disparities in a life-course perspective [Doctoral thesis]. Stockholm: Stockholm University; 2011.

[66] Mayeux L. Effects of popularity and gender on peers’ perceptions of prosocial, antisocial, and jealousy-eliciting behaviors. Merrill-Palmer Q. 2011;57:349–374.

